# Atypical Mycobacterial Infection Arising Amid Corticosteroid Therapy for Livedoid Vasculopathy

**DOI:** 10.1155/2019/1840280

**Published:** 2019-10-07

**Authors:** John R. Edminister, Nicole Dominiak, Lorie D. Gottwald

**Affiliations:** ^1^University of Toledo College of Medicine and Life Sciences, 3000 Arlington Avenue, Toledo, OH 43614, USA; ^2^Department of Pathology, University of Toledo Medical Center, 3000 Arlington Avenue, Toledo, OH 43614, USA; ^3^Department of Dermatology, University of Toledo Medical Center, 3000 Arlington Avenue, Toledo, OH 43614, USA

## Abstract

Patients who suffer from rare skin diseases may try numerous therapies with many potential side effects before achieving remission. Livedoid vasculopathy (LV) is one such rare disease that lacks a definitive treatment as evidenced by randomized controlled trials. Although corticosteroids help reduce the pain flares associated with LV, they come at the risk of immunosuppression. We present a case of disseminated cutaneous infection of *M. chelonae/abscessus* arising in a diabetic patient on long-term corticosteroid therapy. This patient required an intensive antibiotic regimen and potentially lifelong antibiotic suppression pending improvement of her disseminated cutaneous infection. We report this case to increase awareness of the diagnostic consideration of atypical, rapidly growing mycobacterial (RGM) infection when encountering patients with a diffuse onset of ulcerative skin nodules amid a background of diabetes and long-term corticosteroid use.

## 1. Introduction

Livedoid vasculopathy (LV) is a chronic, painful condition that typically affects the cutaneous vasculature of the distal legs [1]. There is no standard or definitive treatment of livedoid vasculopathy as evidenced by a randomized controlled trial, perhaps due to its rare incidence at 1 : 100,000 [2]. Instead, case reports and series have demonstrated a multitude of therapeutic options with the most common being anticoagulants, antiplatelet drugs, systemic steroids, and intravenous immunoglobulins (IVIG) [3]. These therapies each have a wealth of adverse effects and contraindications that can make it difficult to treat a patient with comorbidities alongside a rare disease such as livedoid vasculopathy. Although the pathogenesis of LV is now thought to involve thrombotic occlusion, some patients may fail or have contraindications to the now commonly used anticoagulant/antiplatelet agents. These patients might then find relief with systemic corticosteroids, as some patients using corticosteroids have reported better healing and prompt resolution of pain flares associated with livedoid vasculopathy [4].

It is known that corticosteroids modulate the innate immune system and suppress cellular immunity [5]. Opportunistic infection is an unfortunate development of immunosuppression secondary to corticosteroids. A rare example of this is disseminated cutaneous infection with nontuberculous mycobacterial species such as the *Mycobacterium chelonae–abscessus complex*. These rapidly growing nontuberculous mycobacteria are ubiquitous in environmental soil and water [6] and can cause three different manifestations of disease—catheter infection, local infection of skin or bone, or a disseminated cutaneous infection; the latter being the most common clinical presentation of *M. chelonae* infection [7]. The morphology of disseminated cutaneous lesions include pustules, hyperkeratotic plaques, ulcers with sinus tracts, nodules which may suppurate, and finally a sporotrichoid appearance with proximal spread along lymphatic vessels [8]. In one study, 90% of these disseminated cutaneous infections occurred with corticosteroid therapy, with some underlying conditions including autoimmune disease, renal transplant, and rheumatoid arthritis [7]. Additionally, a retrospective study in 2016 suggested that soft-tissue infection with nontuberculous mycobacterial species were three times overrepresented in diabetic patients when compared to the general population [9].

We present a case of disseminated cutaneous infection of *M. chelonae/abscessus* occurring in a diabetic patient on long-term corticosteroid therapy for her rare livedoid vasculopathy.

## 2. Case Presentation

In July 2006, a 50-year-old female presented to the dermatology clinic with a complaint of painful skin changes in her bilateral lower extremities. She was employed as a dog groomer, and her past medical history was significant for type 2 diabetes mellitus with insulin requirements, gastritis, and severe valvular heart disease affecting the tricuspid, mitral, and aortic valves with surgical repair of the aortic valve. On physical examination, the patient was found to have linear, hyperpigmented macules on the bilateral lower legs with foci of scarring and ulceration. Two biopsies of the proximal and distal left lower leg suggested livedoid vasculopathy pending clinical correlation. She was additionally found to have an elevated antithrombin 3 activity of 124 (reference range 70.0–120.0), which is strongly suggestive of an underlying prothrombotic component to her condition [10]. At this time, therapeutic options for livedoid vasculopathy were considered and offered to the patient.

The patient's preferences and past medical history presented several obstacles to treatment of her LV. She repeatedly refused anticoagulant therapy because her husband had previously had issues with the diet restrictions and INR monitoring mandated by the use of warfarin. Antiplatelet agents were avoided due to her history of severe gastritis that was onset prior to our management of her LV. Intravenous immunoglobulin was considered; however, the patient could not afford the co-pay and her cardiologist recommended against IVIG due to the risk of these hyperosmolar preparations causing fluid overload in this patient with severe valvular heart disease. The patient was eventually started on an acceptable treatment regimen consisting of oral dapsone 100 mg once daily and prednisone 10 mg once daily, with the addition of doxepin or tramadol for intermittent pain control. With these medications, she achieved intermittent remission of her LV for several years.

In 2015, she began having intermittent, painful flares of her LV which were managed by increasing her dapsone to 150 mg once daily and increasing her prednisone to 20 mg. She sometimes required burst doses of 60 mg once daily. Attempts to wean her prednisone back down to 10 mg were rarely successful, and this dosing became an ongoing concern in 2018 when she began having severe hyperglycemic episodes which resulted in a brief hospitalization. Her insulin delivery was also switched to a pump system.

**Figure 1:**
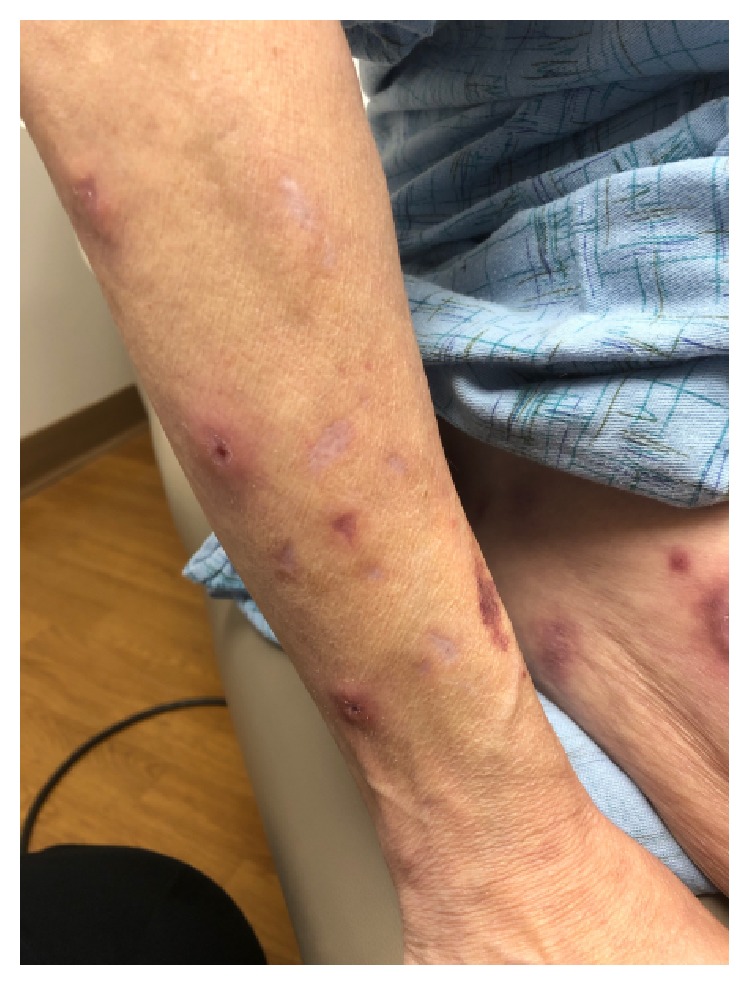
The right dorsal forearm was found to have two firm, tender erythematous papulonodules, one with central ulceration.

**Figure 2:**
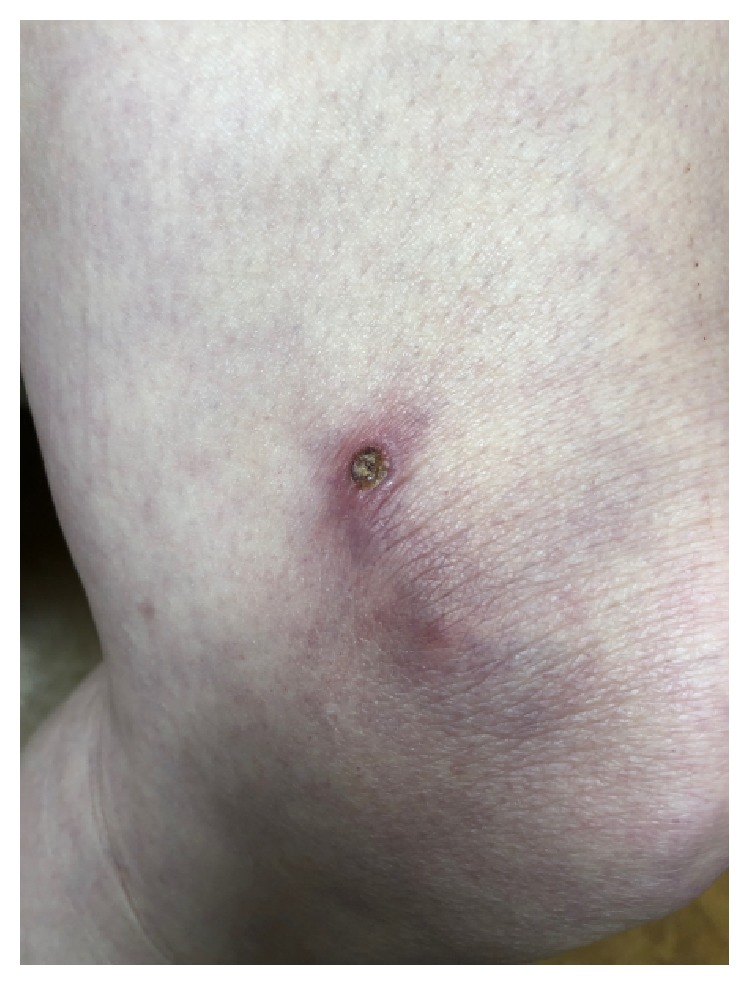
The right lateral thigh was found to have a purpuric patch with central ulceration and necrosis.

**Figure 3:**
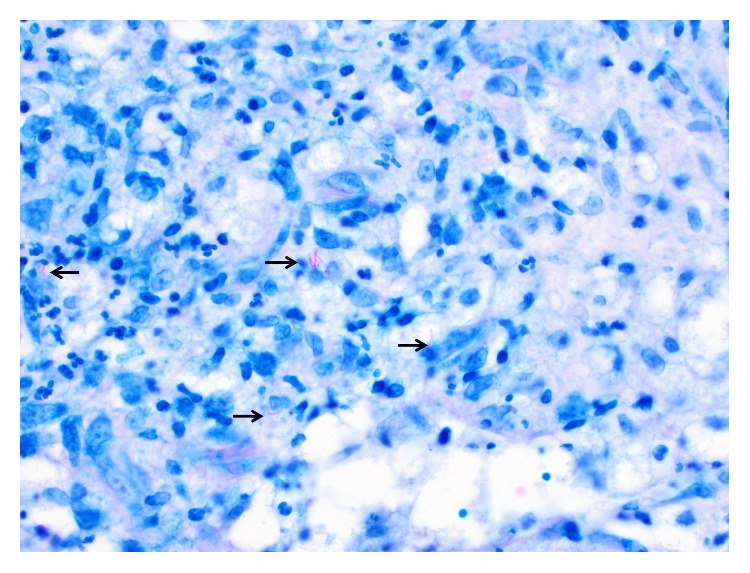
Kinyoun stain (40x). Acid fast bacilli (arrows) within the center of the suppurative granuloma.

In the fall of 2018, she presented to dermatology during an acute LV pain flare and was coincidentally found to have an erythematous papule at the right dorsal forearm, which she attributed to a possible insect bite or a scratch from her dog. Three weeks later, she reported worsening of this right forearm lesion as well as new onset of two painful, ulcerative lesions on her right thigh and right forearm. On examination, the right dorsal forearm was now found to have two firm, tender erythematous papulonodules, one with central ulceration (Figure 1). The right lateral thigh was found to have a single purpuric patch with central ulceration and necrosis (Figure 2). The bilateral forearms additionally had a few scattered linear superficial abrasions consistent with animal scratches. No other new lesions were found on examination. She perceived these lesions as dissimilar from her typical LV, but at this time mycophenolate mofetil 500 mg twice daily was added for her ongoing LV flares. A 4 mm punch biopsy of a right dorsal forearm lesion revealed suppurative, granulomatous inflammation in the deep reticular dermis with demonstration of acid-fast organisms on AFB stain (Figure 3).

The patient was subsequently evaluated for systemic mycobacterial disease. A chest X-ray showed no evidence of pulmonary mycobacterial disease. Laboratory testing was negative for HIV, AFB blood culture, and Tb QuantiFERON. Her CRP was elevated at 13.3, and the CBC revealed a mild leukocytosis of 13.00 with mild neutrophilia of 79.4%, elevated absolute neutrophils (10.3), and absolute immature granulocytes (0.4).

The state Department of Health identified rapidly growing *Mycobacterium abscessus/chelona*e on her right forearm tissue culture. Susceptibility testing and treatment guidance was subsequently provided by a national center specializing in mycobacterial consultation. Susceptibility results revealed resistance to cefoxitin, doxycycline, sulfamethoxazole-trimethoprim, and amoxicillin-clavulanic acid and sensitivity to clarithromycin, azithromycin, linezolid, imipenem, and amikacin. There was also intermediate sensitivity to ciprofloxacin and moxifloxacin.

The physicians of the Dermatology and Infectious Disease departments then coordinated management of her simultaneously flaring livedoid vasculopathy and disseminated cutaneous mycobacterial infection. The patient had a PICC line placed for her antimycobacterial regimen which consisted of oral azithromycin 250 mg once daily, intravenous imipenem 500 mg every 12 hours, and finally intravenous amikacin 12–15 mg/kg on Mondays, Wednesdays, and Fridays. Additional treatment with fluoroquinolones was not considered because she had a previously documented allergic reaction of a blistering skin eruption. This regimen was initially to be continued for a total of 8–12 weeks depending on her response, followed by oral azithromycin monotherapy for at least an additional 6 months. Lifelong suppression with oral monotherapy is also being considered pending her clinical response and tolerance to azithromycin.

At 10 weeks of treatment her physical exam revealed improvement of the ulcers of the right forearm, right thigh, and left foot. However, she also developed three new left knee ulcers consistent in appearance with her other mycobacterial lesions. For this reason, her current regimen is to be continued until her follow-up appointment for PICC line removal at 17 weeks of treatment. At that time, she will transition to daily maintenance therapy of oral azithromycin if she demonstrates adequate improvement in the existing lesions.

Meanwhile, the treatment of her livedoid vasculopathy was optimized by the dermatology team. Her prednisone was tapered down to 10 mg once daily, and both the dapsone and mycophenolate mofetil were discontinued. She has since been started on warfarin 1 mg once daily. This low dose was chosen due to reports of supratherapeutic INR from interaction of warfarin and azithromycin [11]. Her LV was found to be stable at her follow-up appointment 9 weeks after initiating and continuing the same dose of warfarin.

## 3. Discussion

Livedoid vasculopathy is a relatively rare condition with an incidence of 1 : 100,000 at a 3 : 1 ratio of women to men [2]. The clinical manifestations of early LV classically involve the bilateral distal legs and ankles with a reticular pattern of purple plaques and papules which intermittently flare into painful ulcers; late manifestations include stelliform, porcelain-white scarring known as “atrophie blanche” [1]. Because LV is not a true inflammatory vasculitis but rather a thrombotic vasculopathy, clinical suspicion must be confirmed with histopathologic evidence demonstrating dermal vascular changes such as segmental hyalinosis, thrombosis, and fibrin deposition [2].

This case exemplifies the struggles and complications that patients with rare skin diseases may encounter. There is no definitive treatment for livedoid vasculopathy, and in our case the preferred treatments were either contraindicated or deferred due to patient preference. This resulted in her being treated with dapsone and prednisone for years. The use of corticosteroids in LV is not unheard of, as steroids were the second-most prescribed drug for LV behind heparin in one pre-study population [10]. However, that same cohort study found approximately 12 patients with LV to have no better than a moderate response to steroids. On the other hand, Feng et al. described 22 patients with LV on combined regimens that included steroids, and they found that steroids were effective in hastening resolution of acute, ulcerative LV flares [12]. Our patient similarly responded well to increasing doses of prednisone during her acute LV flares. However, this treatment came with the well-known adverse effect of immunosuppression. Her diabetes and recent hospitalization for hyperglycemia were additional concerning factors in the management of this patient with corticosteroids.

This patient's development of a rapidly growing nontuberculous mycobacterial skin infection can be attributed to multiple factors including her corticosteroid therapy, ongoing poorly controlled diabetes mellitus, and a possible direct inoculation or environmental exposure. The most common presentation of disseminated RGM skin infections is with an underlying corticosteroid exposure or defective cellular immunity [8]. A case report in 2015 describes a patient who developed a disseminated cutaneous *M. chelonae* infection amid systemic corticosteroid and mycophenolate mofetil treatment for systemic lupus erythematosus [13]. Our patient did have a brief trial of mycophenolate mofetil for her LV flares. However, this was initiated after her initial right forearm lesion was first observed and it was discontinued when the patient began her antibiotic regimen for the confirmed RGM infection. Therefore, we do not believe mycophenolate mofetil played a role in the development of our patient's infection.

Diabetics have also been demonstrated to have increased tendency for developing RGM infections, and some case reports trace atypical mycobacterial infections to an inoculating event with unclean or improperly used insulin pens or syringes [14–16]. Other local inoculating events have been reported in the literature, including tattooing and surgical procedures such as liposuction and liposculpture [17, 18].

Our patient was interviewed regarding possible events surrounding the onset of her infection. Although one early lesion was on her right lateral thigh, she denied any known insulin injections at that site prior to onset of her infection. She denied recent falls, penetrating trauma, surgeries, cosmetic procedures, biopsies, tattoos, or injections besides the previous use of her insulin pens and glucometer. Of note, she was working as a part-time pet groomer and frequently bathed dogs in water. She also owned several dogs and cats and reported being frequently scratched on the forearms and legs by these animals.

The zoonotic potential of several mycobacterial species is well-documented, ranging from bovine tuberculosis to fish tank granuloma primarily caused by *Mycobacterium bovis* and *Mycobacterium marinum*, respectively [19, 20]. Zoonotic transmission of *Mycobacterium microti* infection by cats, racoon dogs, and mice has been suggested; however, like *M. bovis* and *M. marinum*, the *M. microti* species is a slow-growing mycobacterial species [21]. A search of medical and veterinary literature did not reveal any known cases of zoonosis involving the *M. chelonae* complex. Although our patient's profession as a dog groomer is intriguing, we suspect that she likely encountered her infection through a common environmental source of RGM such as water or soil.

Disseminated cutaneous infection with rapidly growing nontuberculous mycobacteria may result in significant morbidity due to the prolonged therapeutic timeline that typically lasts a minimum of 12 months [13]. The *M. abscessus* species is resistant to conventional antimycobacterial drugs and those that are usually effective against other RGM such as erythromycin and minocycline. The initial choice for local disease can be oral monotherapy with clarithromycin; however, disseminated cutaneous disease requires an oral agent (such as clarithromycin or azithromycin) with parental combination involving tobramycin, imipenem, and amikacin [16]. We consulted a national center for mycobacterial susceptibilities and treatment guidance, and we agreed upon a regimen with one oral agent and two parenteral agents administered via PICC line at home for a minimum of four months. This would be followed by potentially lifelong oral monotherapy with azithromycin. Her PICC line requirement has known risks including luminal occlusion, venous thrombosis, and local or systemic infection [22]. Our patient is now retired and has a good support system in place with her husband; however, she does require a visiting home nurse to aid with her PICC line.

Due to concerns of adverse effects of her antimicrobial regimen, our patient required weekly labs including complete blood count, basic metabolic panel, and serum amikacin levels. Of particular note was a time of 10 weeks into her treatment when amikacin was temporarily discontinued due to a concerning creatinine elevation of 1.62 compared to her baseline of 1.17 prior to initiating antibiotic therapy.

## 4. Conclusion

This case demonstrates the frustrations that both patients and dermatologists experience when treating a rare skin disease in a patient with multiple comorbidities. We report this case to increase awareness of the diagnostic consideration of atypical mycobacterial infection, especially when encountering patients with a diffuse onset of ulcerative skin nodules amid a background of immunosuppression secondary to long-term corticosteroid use and diabetes.
